# Bisphenol A: Where to Now?

**DOI:** 10.1289/ehp.12492

**Published:** 2009-03

**Authors:** John R. Bucher

**Affiliations:** NTP, NIEHS, National Institutes of Health Department of Health and Human Services, Research Triangle Park, North Carolina, E-mail: bucher@niehs.nih.gov

Recently, *Time* magazine published an article titled “The Year in Medicine: From A to Z” (Park et al. 2008). The letter “B” was represented by the controversy over bisphenol A, a ubiquitous chemical used in polycarbonate and polyvinyl chloride plastics and epoxy resins and found in the urine of > 90% of Americans. The debate over whether bisphenol A poses a threat to human health has been brewing for the better part of the past decade.

On 3 September 2008, the National Toxicology Program (NTP) Center for the Evaluation of Risks to Human Reproduction (CERHR) weighed in by releasing a report that significantly contributes to this ongoing discussion. The *NTP-CERHR Monograph on the Potential Human Reproductive and Developmental Effects of Bisphenol A* (NTP 2008a) identified evidence from experimental animal studies that raised “some concern” that current levels of exposure to human fetuses, infants, and children may result in developmental changes in the prostate gland and brain and diminish sexually dimorphic behaviors. “Some concern” represents the mid-point of a five-level scale of concern used by the NTP that ranges from “negligible” to “serious” concern. A lower level, “minimal concern,” was also expressed for possible changes in development of the mammary gland and an earlier age of attaining puberty in females.

The NTP’s opinion on the level of concern for effects of bisphenol A on human reproduction and development stemmed from a 2-year analysis of a very limited number of available human studies but nearly 1,000 studies in experimental animals. Many of the laboratory studies explored effects on offspring of pregnant rodents receiving “low doses” of bisphenol A (< 5 mg/kg body weight/day, and including studies performed with much lower doses) during critical periods of development. The NTP Board of Scientific Counselors (2008) provided peer review and suggestions for refinement of the NTP CERHR’s conclusions (NTP 2008a), and the Science Board to the Food and Drug Administration (FDA 2008a, 2008b) also expressed agreement with the evaluation.

The NTP’s evaluation of bisphenol A expressed “some concern” because many of the developmental effects reported in laboratory animals were observed at exposures to bisphenol A similar to those experienced by humans. Collectively, the findings could not be dismissed. Similar conclusions were reached by Health Canada (2008) and by participants at a workshop examining the potential relationship between bisphenol A and negative trends in human health (vom Saal et al. 2007). However, the NTP CERHR report (NTP 2008a), as well as other reviews, identified many areas of uncertainty and data gaps that should be addressed to fully understand bisphenol A’s potential to harm human development.

In the months since release of the NTP-CERHR report (NTP 2008a), the literature on exposures and potential human health effects of bisphenol A has continued to grow (Calafat et al. 2008; Hugo et al. 2008; Lang et al. 2008; Leranth et al. 2008), raising public concern and generating more questions. Lists of research needs have been assembled (NTP 2008a; vom Saal et al. 2007). The NTP and the National Institute of Environmental Health Sciences (NIEHS) Division of Extramural Research and Training (DERT) recently issued a request for information (RFI) to the scientific community seeking information to help focus future research and testing activities (NTP 2008b). The RFI seeks information about *a*) on going research on the health effects of bisphenol A; *b*) unmet research needs; and *c*) suggestions for collaboration and cooperation between investigators to improve efficiency and timeliness in filling the information gaps. Together, the NTP and DERT will carefully consider the responses to this RFI as we develop research programs and explore other ways to address these issues in the future.

The RFI (NTP 2008b) listed a number of general topics that scientists have consistently raised as areas where research is needed: *a*) the need to better understand sources of human exposures; *b* ) the need to compare the metabolism of bisphenol A among rodents, nonhuman primates, and humans and understand how it changes with age; *c*) the need for physiologically based pharmaco kinetic (PBPK) models to provide a scaffold for quantitatively assessing the consistency of outcomes across studies performed with widely different doses and designs; and *d*) the need for additional developmental toxicology studies of traditional design and power, but with modifications to provide the capability to detect the range of effects reported in academic studies as well as functional consequences as the animals age.

The NTP has begun work in several areas. In collaboration with the Centers for Disease Control and Prevention and academic investigators, we are facilitating an evaluation of exposures to bisphenol A in infants in neonatal care settings and in children < 6 years of age. Together with the FDA National Center for Toxicological Research, we have initiated studies to obtain the data for constructing PBPK models in rodents and nonhuman primates, and we are planning studies to explore the long-term consequences of perinatal exposure to bisphenol A in order to understand the potential impact to humans of the developmental changes reported in numerous laboratory animal studies. The NTP and DERT are considering a number of strategies to provide the academic community access to the animals and tissues generated in these studies. We will provide additional details on the status and direction of our bisphenol A testing program through public meetings of the NTP Board of Scientific Counselors.

Collectively, the results of these studies should begin to chip away at the uncertainties and research gaps and provide a better perspective of the potential threat that exposure to bisphenol A poses to public health.

## Figures and Tables

**Figure:**
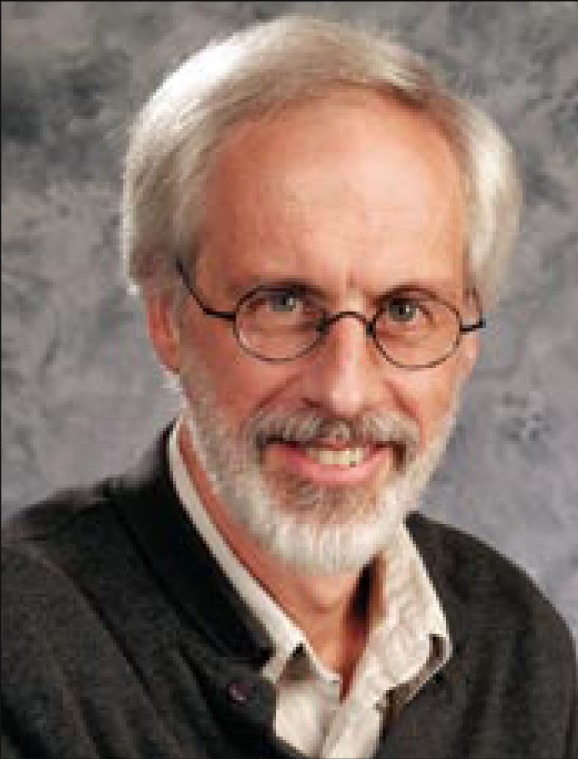
John R. Bucher
